# Wireless Metal Detection and Surface Coverage Sensing for All-Surface Induction Heating

**DOI:** 10.3390/s16030363

**Published:** 2016-03-11

**Authors:** Veli Tayfun Kilic, Emre Unal, Hilmi Volkan Demir

**Affiliations:** 1 Department of Electrical and Electronics Engineering, Department of Physics, UNAM-Institute of Materials Science and Nanotechnology, Bilkent University, Ankara TR-06800, Turkey; vkilic@bilkent.edu.tr (V.T.K.); unale@bilkent.edu.tr (E.U.); 2 School of Electrical and Electronic Engineering, School of Physical and Mathematical Sciences, Nanyang Technological University, Singapore 639798, Singapore

**Keywords:** electromagnetic induction, metal detection, wireless sensing, all-surface heating, coils

## Abstract

All-surface induction heating systems, typically comprising small-area coils, face a major challenge in detecting the presence of a metallic vessel and identifying its partial surface coverage over the coils to determine which of the coils to power up. The difficulty arises due to the fact that the user can heat vessels made of a wide variety of metals (and their alloys). To address this problem, we propose and demonstrate a new wireless detection methodology that allows for detecting the presence of metallic vessels together with uniquely sensing their surface coverages while also identifying their effective material type in all-surface induction heating systems. The proposed method is based on telemetrically measuring simultaneously inductance and resistance of the induction coil coupled with the vessel in the heating system. Here, variations in the inductance and resistance values for an all-surface heating coil loaded by vessels (made of stainless steel and aluminum) at different positions were systematically investigated at different frequencies. Results show that, independent of the metal material type, unique identification of the surface coverage is possible at all freqeuncies. Additionally, using the magnitude and phase information extracted from the coupled coil impedance, unique identification of the vessel effective material is also achievable, this time independent of its surface coverage.

## 1. Introduction

**Figure 1:**
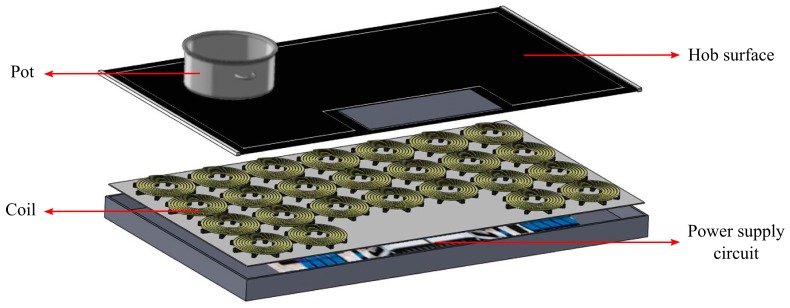
Conceptual view of an all-surface induction heating system structure, where system parts and the pot are illustrated with red arrows.

Although the idea of power transmission via electromagnetic waves goes back to the early 20th century [1], induction systems have been most recently becoming increasingly more popular and crucial and their usage areas have been expanding because of their high efficiency and safety [2,3]. Today, in addition to melting, metal processing and wireless charging, induction cooking is one of the most important applications of induction systems. In induction hobs, coils are placed beneath the hob surface. These coils are fed by a supply circuit with an alternative current of frequency between 20 KHz and 100 KHz in general. Circulating currents on the coils produce magnetic fluxes and these magnetic fluxes couple to a vessel located on the hob surface. Therefore, in induction hobs, instead of conduction heat transfer, energy is transferred wirelessly from a coil to a vessel via electromagnetic fields. The transferred electromagnetic energy turns into heat on the vessel surfaces as a result of the induced eddy currents [3,4,5,6]. Accordingly, induction hobs are more efficient, non-hazardous and cleaner for users compared to conventional heaters including resistive and flame heaters [6]. In regular induction hobs, vessels should typically have similar sizes with those of coils and they should be placed right on top of a coil for efficient heating. To overcome these issues, induction hobs with an array of small-area coils have been proposed [7,8,9]. Thanks to the array structure, efficient heating of vessels with different shapes and sizes located anywhere on the hob might be possible. Therefore, a very recent important implementation in induction hobs is all-surface heating [10,11]. Structure of an all-surface induction heating system is visualized in Figure 1. 

In all-surface induction hobs, location of the vessel should be identified together with its size in order to power up the corresponding coils for efficient, safe, and low-cost heating. In commercial induction hobs, pan detection is made using the damping factor measurement [12,13,14]. In these systems, if a coil is not loaded, then long oscillation signals are detected. On the other hand, if the coil is loaded with a metal vessel, then a few-cycle damped oscillation signal is observed. This method is useful for detecting the vessel presence. However, this approach cannot specify the vessel’s exact position on the surface of the hob or its material.

Since an all-surface induction hob is a radically new topology, there are no previous reports in the literature addressing the problem of metal coverage sensing to identify which coils to be powered up in all-surface induction for efficient heating. In this work, we propose and demonstrate a simple and robust solution that provides us with the ability to detect the vessel presence while uniquely sensing its surface coverage and identifying its effective material type at the same time in all-surface induction heating systems. Here, we systematically study variations in the inductance and resistance of an all-surface induction coil coupled to a metal plate at different locations with different coverage areas. In this study, we obtain maps of the magnitude and angle variations in the coupled system’s impedance. Although some previous reports discuss variations of inductance and resistance of a coil in an induction heater for heating measurements [15,16], this work is the first account of the proof-of-concept demonstration of wireless metal detection and surface coverage sensing independent of the metallic vessel material type in all-surface induction heating, which is essential to determining the vessel coverage by each coil for powering and obtaining the information of the vessel material and size for efficient heating.

## 2. Experiments

**Figure 2:**
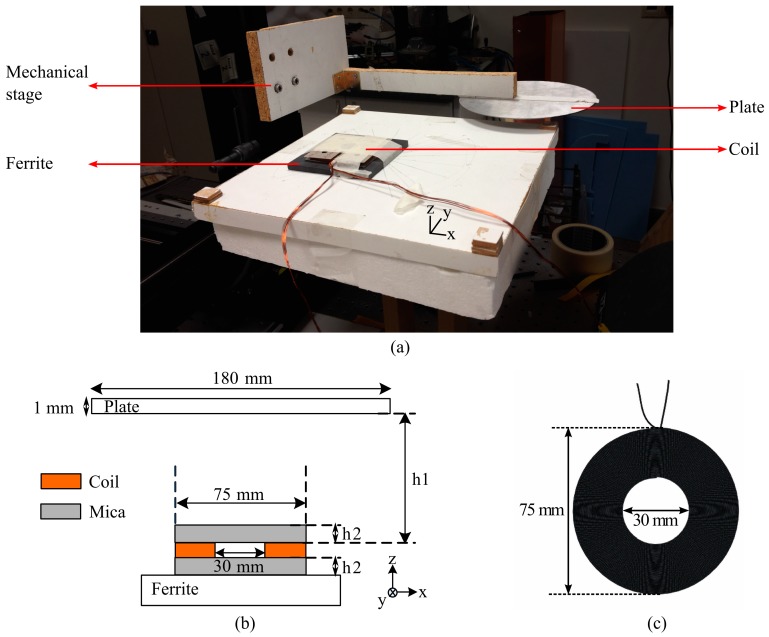
(**a**) measurement setup and its parts, where components are pointed out and indicated with red arrows; (**b**) coupled system from side-view together with its geometrical parameters; and (**c**) the coil structure used in measurements and its geometrical sizes.

In our experimental study, a plate was used to be placed over a conventional circular coil and moved around horizontally without changing its perpendicular distance to the coil. Inductance and resistance values of the studied coil were measured for each position of the plate at two different frequencies (20 and 100 KHz) (by means of an LCR meter Agilent 4263B). Measurement setup is shown in Figure 2a. In experiments while the coil was immobile, the plate was systematically shifted on the *x*-*y* plane with the help of a computer-controlled mechanical stage. As a result, the changes of inductance and resistance values with an area of the coil covered by the plate were measured. 

A side-view of coupled system is sketched with its geometrical parameters in Figure 2b. As represented in the figure, the coupled system is constructed by a coil, a plate and a ferrite layer below the coil. The plate is a disk with a diameter of 180 mm and a thickness of 1 mm. This diameter of this plate is in the standard range of vessel dimensions, and it is selected to be larger than that of the coil to model coil array heating in all-surface application. The distance between the coil and the plate (h1) was set to 8 mm, which is similar to the typical thickness of a glass ceramic surface in conventional hobs. During characterization, the plate was scanned across the planar surface 8 mm above the coil. Similarly, the distance between the ferrite layer and the coil (h2) was set to 0.4 mm. This is because of the substrate (mica) layer used in the manufacturing of the coil. Another mica layer exists on top of the coil as well, but its thickness was included in h1.

On the other hand, the coil used in these measurements has a conventional circular shape with a 75 mm outer diameter and a 30 mm inner diameter. The outer diameter was set to be the same with the minimum vessel dimension (e.g., a coffee pot) accepted by manufacturers. The coil consists of 44 circular turns in total and, in each turn, 13 copper wires with 0.25 mm diameter exist. Coil structure is shown in Figure 2c. These coils with the same structure were used in our other studies, too [17].

Because vessels used in daily life are made of various materials, measurements were repeated with stainless steel (AISI 430) and aluminum plates. These materials were selected due to their opposite properties effective on the induction heating process. In contrast to aluminum’s very low magnetic permeability and electrical resistivity, stainless steel has huge permeability and relatively high resistivity. Although aluminum is not a proper vessel material to be used in induction heating, stainless steel is one of the most appropriate vessel materials. Therefore, the measurements were done with one of the most and least proper vessel materials.

## 3. Results and Discussion

**Figure 3:**
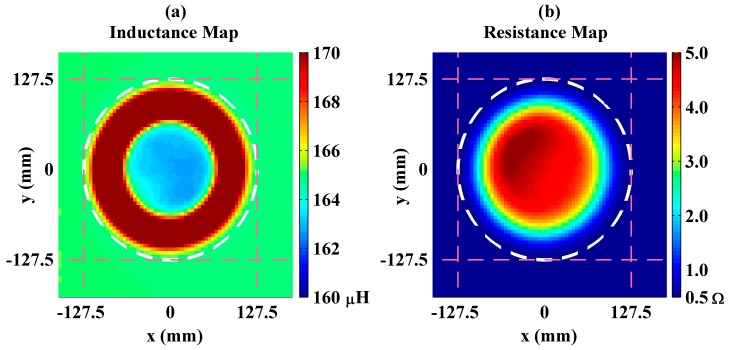
Measured inductance (**a**) and resistance (**b**) maps for the system where the coil is loaded by the steel plate as a function of its lateral position at a horizontal plane 8 mm above the coil. Here, the coil is centered at the (0,0) origin. In addition, the white dash circles represent the points where the coil and the plate projections are tangential. End points of the circles are pointed out with pink ticks at −127.5 mm and 127.5 mm on the *x* and *y* axis.

First, the measurements were obtained using a stainless steel plate. Mappings of the system’s inductance and resistance values while the plate is scanned across at 20 KHz are depicted in Figure 3. In the figure, each point represents the center-to-center distances between the plate and the coil projections on the plane and the color bar data correspond to the measured resistance and inductance values. 

Inductance of the unloaded coil is around 165 μH (see Figure 3a). However, as the plate and the coil centers get closer, the system’s equivalent inductance first increases and then decreases. Normally, monotonic decreasing with coverage area is expected because of enhancement in coupling. The reason for unexpected inductance increase is induced currents on the top surface of the plate that are in reverse direction with currents on the bottom surface. In the case where the coil is partially covered, looping magnetic fields produced by the coil create such reverse currents on the plate’s top surface. In addition, bending of the induced currents on the bottom surface of the plate causes reverse currents. Since stainless steel has a huge permeability, the magnetic flux produced by the coil induces eddy currents not on the plate’s top surface but on its bottom surface. However, as the plate moves out of the coil’s projection, the induced currents on the bottom surface goes through the plate’s side edges and flows on the top surface as well. These currents on the bottom and top surfaces are in reverse directions and thus they construct a complete loop. The induced current distribution on the steel plate’s bottom and top surfaces were calculated using 3D full electromagnetic solutions given in supplementary materials (see Figures S1–S3). On the other hand, resistance of the unloaded coil is approximately 0.5 Ω and increases monotonically with the plate’s coupling (see Figure 3b). This is because of the fact that the induced eddy currents on the plate turn into heat and dissipate power independently from its direction. Moreover, in the figures it is observed that, in spite of their circular distribution, the inductance and the resistance plots are not purely symmetric. The reason for this measurement error is that the plate was not perfectly parallel to the coil plane.

**Figure 4:**
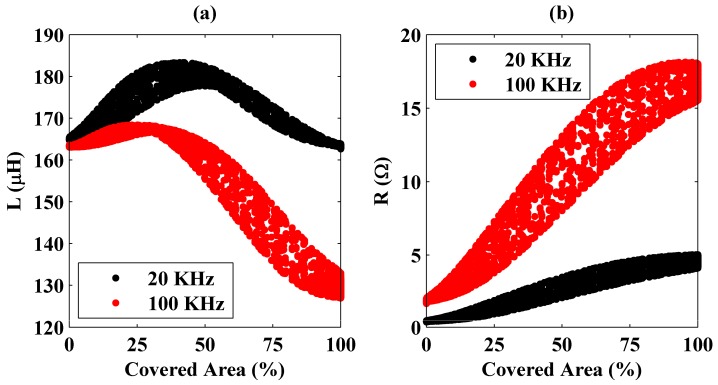
Variation of the measured inductance (**a**) and resistance (**b**) as a function of the coil area covered by the steel plate at 20 and 100 KHz frequencies.

Measurements were repeated at 100 KHz frequency. It was observed that the inductance values are lower at 100 KHz than those observed at 20 KHz. On the other hand, it was also seen that the system equivalent resistance increases with frequency. One of the effects for the enhancement in resistance is skin depth. Since the skin depth decreases with operational frequency, the resistance increases. These observations are clearly seen in Figure 4 where changes of the inductance and resistance values with a percentage area of the coil covered by the plate are presented.

The percentage area is simply ratio of the coil area covered by the plate to the coil’s total area. The total area and the partial area covered by the plate are calculated by Equation (1) [18].

(1) $$\begin{array}{c} A_{\mathrm{coil}}=\pi r^{2}\\ {Α}_{\mathrm{cov}\mathrm{ered}}=\;\left\{ \begin{array}{ll} 0 & ,r+R<d\\ \pi r^{2} & ,d<R-r\\ r^{2}\cos^{-1}(\frac{d^{2}+r^{2}-R^{2}}{2\mathrm{dr}})+R^{2}\cos^{-1}(\frac{d^{2}+R^{2}-r^{2}}{2\text{dR}})-\frac{1}{2}\sqrt{\left[{\left(d+r\right)}^{2}-R^{2}\right]\left[R^{2}-{\left(d-r\right)}^{2}\right]} & ,R-r\leq d\leq r+R \end{array}\right. \end{array}$$

Here, A_coil_ and A_covered_ are the coil’s total area and the intersection area between the coil and the plate’s projection, respectively. In addition, d is the distance between the centers of the plate (with radius R) and the coil (with radius r). 

In the figure, it is seen that the system’s equivalent resistance has a trend of monotonically increasing, but the same behavior is not held for the inductance. In addition, variations in the inductance and resistance are higher at 100 KHz than those obtained at 20 KHz. Therefore, it can be deduced that, for the steel plate’s detection, the resistance should be considered rather than the inductance, and it is easier at high operation frequencies. In addition, in the figures, there exist multiple inductance and resistance values that correspond to constant covered area percentages. As explained previously, the reason for this measurement error is the plate’s not being completely parallel to the coil.

**Figure 5:**
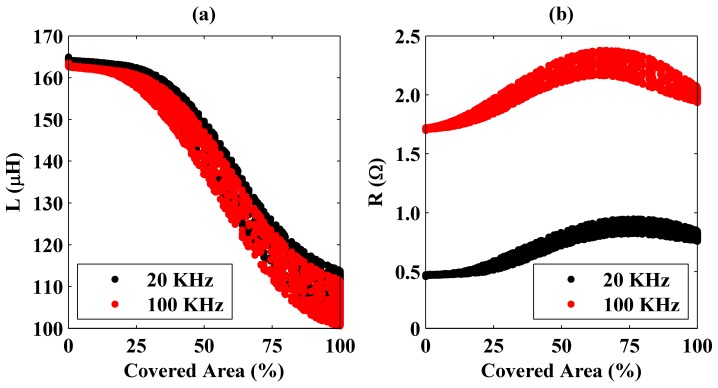
Variation of the measured inductance (**a**) and resistance (**b**) as a function of the coil area covered by the aluminum plate at 20 and 100 KHz frequencies.

Similar measurements were conducted using an aluminum plate whose geometry is the same with that of the steel plate. Variations of the inductance and the resistance values with the coil area covered by the aluminum plate are shown in Figure 5.

In the figure, a behavior different from that observed with the steel plate is expressed. Here instead of the resistance, system’s inductance has a monotonically decreasing trend with the covered area. Therefore, for the aluminum plate’s coverage detection, the inductance value should be considered rather than the resistance but high operation frequencies do not make this procedure easier. In Figure 5, the inductance does not change considerably with frequency. This is because of low magnetic permeability of the aluminum at 20 KHz and 100 KHz frequencies. Magnetic flux produced by the coil couples less to the aluminum plate.

These different behaviors observed for the steel and the aluminum plates are because of their magnetic and electrical properties. For the aluminum plate (see Figure 5b), the measured resistance first increases and then decreases as the coil moves out of the plate’s projection. Small increases initially observed are due to the plate’s edges. Currents induced on the aluminum plate encounter discontinuities at the edges, which increases the equivalent resistance. However, at some point (at around 75% at 20 KHz and 60% at 100 KHz in Figure 5), the resistance decreases towards the level of unloaded coil’s resistance. This is because of the fact that, after some point, decrease in coupling between the coil and the plate dominates the edge effects. Since the aluminum’s resistivity is very low, this effect is seen here, but it is negligible for stainless steel because of its high resistance.

Moreover, the measured inductance has a monotonically decreasing trend, which is different from that observed previously in Figure 4 for the steel plate. Because of its low magnetic permeability, the induced currents on the bottom surface of the aluminum plate couple to the top surface considerably. In other words, because of the coil’s magnetic fluxes, currents are induced on both bottom and top surfaces of the plate. These currents flow in the same direction. Although there still exist reverse currents on the top surface as a result of bending of the currents on the bottom surface near the plate edges and the looping magnetic fields, the total current on the top surface is in the same direction with the current on the bottom surface. Therefore, a continuous decrease in the measured inductance is observed with the covered area. Current distributions on the aluminum plate located at different positions were also calculated using 3D full electromagnetic solutions shown in supplementary materials (see Figures S4–S6).

As a result, by applying small signals at two different frequencies and measuring the system inductance and resistance, the fractional area of the coil covered by the vessel can be determined precisely. However, in all-surface heating, coils are located in an array architecture. To investigate its effect, another circular coil was brought near the coil that is connected to the LCR meter. As expected, since measurements were obtained with small signals and the second coil’s tips were open circuit, the inductance and resistance values were the same within those of the single coil measurements. Therefore, by applying small signals to each of the array coils individually at two different frequencies, in all-surface heaters, the location and the material of a vessel can be uniquely identified. To avoid a large database in the identification process, materials that have similar properties can be grouped together. Furthermore, measured data directly can be used to calculate power transmission from coils to the vessel. On the other hand, algorithms based on the coils’ coverage can be created for easy determination of the vessel’s location. For instance, the center of a vessel can be found by considering the coils’ coverage by the vessel, which are placed side by side. By using distances of these coils to the identified center point, the vessel’s size can be determined together with its approximate shape. 

Since the inductance and resistance values cannot be measured directly from the coil’s small signal response and their calculations require additional time and effort for the system, investigating other parameters that are directly measurable might be a better idea. To this end, we calculated magnitude and angle values of the coil’s impedance from the measured inductance and resistance using Equations (2) and (3), respectively. The magnitude and angle of the coil impedance can be found directly by the amplitude ratio of signal's voltage and current levels that is sent to the coil and the phase difference between them, respectively.

(2) $$\mathrm{abs}\left(Z\right)=\mathrm{abs}\left(R+j\omega L\right)=\sqrt{R^{2}+\omega^{2}L^{2}}$$

(3) $$\mathrm{angle}\left(Z\right)=\mathrm{angle}\left(R+j\omega L\right)=\tan^{-1}\left(\frac{\omega L}{R}\right)$$

Here, Z is the coil’s impedance. Its magnitude and angle are represented with abs(Z) and angle(Z), respectively. Similarly, L and R denote the measured inductance and resistance values, respectively. In addition, ω is the angular frequency.

**Figure 6:**
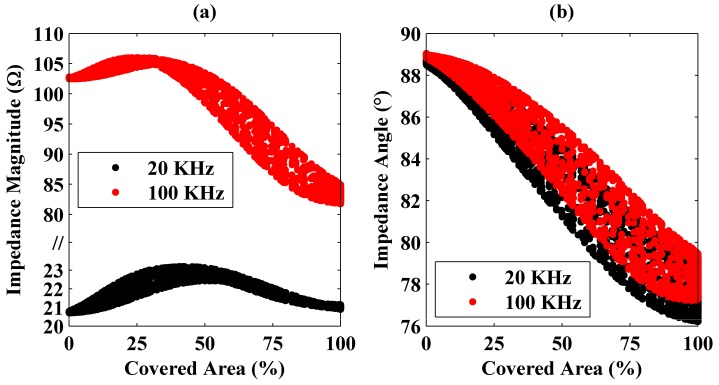
Changes of the impedance magnitude (**a**) and angle (**b**) at 20 and 100 KHz frequencies as a function of the coil area covered by the steel plate. Here, in part (**a**), the *y* axis is scaled nonuniformly for better representation of the calculated magnitudes at different frequencies.

Changes in the magnitude and angle of the coil’s impedance as a function of the steel plate’s loading are shown in Figure 6.

**Figure 7:**
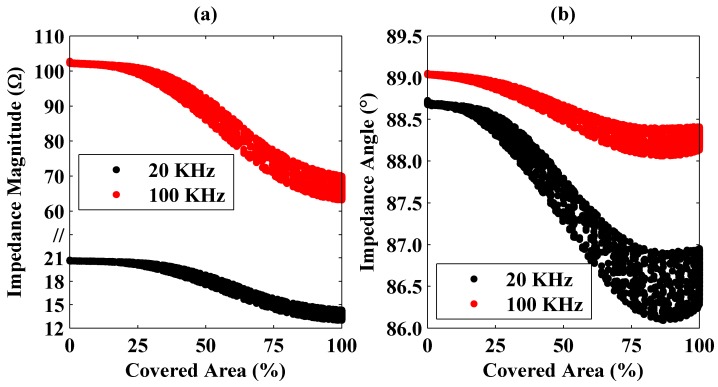
Changes of the impedance magnitude (**a**) and angle (**b**) at 20 and 100 KHz frequencies as a function of the coil area covered by the aluminum plate. Here, in part (**a**), the *y* axis is scaled nonuniformly for better representation of the calculated magnitudes at different frequencies.

Similar plots are given in Figure 7 for the aluminum plate.

From the figures, it is seen that the coverage of a plate and its material can be identified uniquely from the magnitude and angle of the coil’s impedance. In addition, the impedance measurements should be carried out at different frequencies. Otherwise, identification would be hard especially for small covered area percentages because of the fact that impedance magnitudes are similar for both the steel and the aluminum plates while the impedance angles vary in small ranges at a single frequency.

## 4. Conclusions

In this work, a new wireless detection methodology for sensing metal coverage and identifying its material type in all-surface induction hobs is presented. Results show that, for a coupled system, one can determine accurately the partial area of the coil covered by the vessel from the inductance and resistance variation. Material properties of the vessel can also be identified. However, wireless detection is easier at higher frequencies because variations in the inductance and resistance increase with operating frequency.

Moreover, metal detection is also presented here using the amplitude and angle of the coil’s impedance. By measuring voltage and current of each coil sequentially, identification of the plate’s coverage by coils together with its material is possible in all-surface heaters. In addition, impedance measurements at different frequencies make this process easier. These results are beneficial for detecting vessels and determining which coils to power up in all-surface induction hobs.

## Supplementary Materials

The following document is available online at http://www.mdpi.com/1424-8220/16/3/363/s1, Supplementary Materials.doc.

## Abbreviations

The following abbreviation is used in this manuscript:

AISI: American Iron and Steel Institute

## References

[1] Tesla’s Tower—Amazing Scheme of the Great Inventor to Draw Millions of Volts of Electricity Through the Air From Niagara Falls and Then Feed It Out to Cities, Factories and Privat Houses from the Tops of the Towers without Wires. http://www.teslasociety.com/tesla_tower.htm.

[2] Lucia O., Acero J., Carretero C., Burdio J.M. (2013). Induction heating appliances: Toward more flexible cooking surfaces. IEEE Ind. Electron. Mag..

[3] Moreland W.C. (1973). The induction range: Its performance and its development problems. IEEE Trans. Ind. Appl..

[4] Acero J., Alonso R., Burdio J.M., Barragan L.A., Puyal D. (2006). Analytical equivalent impedance for a planar circular induction heating system. IEEE Trans. Magn..

[5] Acero J., Alonso R., Burdio J.M., Barragan L.A., Puyal D. (2006). Frequency-dependent resistance in Litz-wire planar windings for domestic induction heating appliances. IEEE Trans. Power Electron..

[6] Lucia O., Maussion P., Dede E.J., Burdio J.M. (2014). Induction heating technology and its applications: Past developments, current technology, and future challenges. IEEE Trans. Ind. Electron..

[7] Leidig K., Herzog M. (2012). Induction cooking hob. European Patent Specification.

[8] Fournier D., Merliot E., Roux A. (2010). Assembling module of induction coils of a induction heating cooking area and cooking area including the said modules. European Patent Specification.

[9] Roux A. Induction device comprising multiple individual coils for induction heating plates. U.S. Patent Application.

[10] Lucia O., Sarnago H., Burdio J.M. (2015). Soft-stop optimal trajectory control for improved performance of the series-resonant multiinverter for domestic induction heating applications. IEEE Trans. Ind. Electron..

[11] Sanz F., Saguez C., Llorente S. (2015). Induction heating appliance with a mobile double-coil inductor. IEEE Trans. Ind. Appl..

[12] Essig W., Bogdanski F., Fettig G., Horn J. (1995). Inductive based cooking system. U.S. Patent.

[13] Tulu M.E., Yildirim D. Induction cooker design with quasi resonant topology using jitter drive method. Proceedings of the International Conference on Environment and Electrical Engineering (EEEIC).

[14] Holtek Semiconductor Inc. Using the HT45R38 for Pan Detection in Induction Cookers. http://www.holtek.com.tw/english/tech/appnote/uc/pdf/ha0135e.pdf.

[15] Franco C., Acero J., Alonso R., Sagues C., Paesa D. (2012). Inductive sensor for temperature measurement in induction heating applications. IEEE Sens. J..

[16] Jimenes O., Lucia O., Barragan L.A., Navarro D., Artigas J.I., Urriza I. (2013). FPGA-based test-bench for resonant inverter load characterization. IEEE Trans. Ind. Inform..

[17] Kilic V.T., Unal E., Gonendik E., Yilmaz N., Demir H.V. (2016). Strongly coupled outer squircle-inner circular coil architecture for enhanced induction over large areas. IEEE Trans. Ind. Electron..

[18] Weisstein E.W. Circle-Circle Intersection. http://mathworld.wolfram.com/Circle-CircleIntersection.html.

