# Supplementary material for: Wireless Metal Detection and Surface Coverage Sensing for All-Surface Induction Heating
Source: Sensors (Basel). 2016 Mar 11;16(3):363. doi: 10.3390/s16030363 (PMC4813938; doi:10.3390/s16030363)
Supplement: Supplementary file 1 [file sensors-16-00363-s001.pdf]

Supplementary Materials: Wireless Metal Detection and Surface Coverage Sensing for All-Surface Induction Heating

Veli Tayfun Kilic, Emre Unal and Hilmi Volkan Demir

To understand the unexpected non-monotonic behaviors observed for inductance and resistance measurement data, 3D electromagnetic simulations were conducted for both steel and aluminum plates at 20 KHz frequency. For fair comparisons with measurements, 1 V small amplitude voltage source was connected to coil. Tangential current distributions on the bottom and top surfaces of the plates are shown in the following figures.

In Figure S1 it is clear that the induced currents on the bottom surface do not couple to top surface. On the other hand, in Figure S2, the induced currents on the bottom surface goes through the plate side edges and flows on the top surface as well. As we explained in the manuscript, these currents flow in the reverse directions to form a complete loop. Moreover, in Figure S3, the same phenomenon is observed, too. In the figure, additional currents on the top surface that are due to looping magnetic fields produced by the coil are also seen. These currents are in reverse direction with those induced by the coil's produced magnetic flux on the bottom surface. Since in Figure S3, most of the coil is uncovered by the plate, the latter currents have more effects. In the figure to observe currents more clearly, the color bar limit is decreased to 10^5 A/m^2.

Different from Figure S1, in Figure S4 it is seen that the induced currents on the bottom surface couples to top surface in considerable amounts. As we explained in manuscript, this is because of low magnetic permeability of aluminum. Also, in Figure S5 additional currents around the plate edges on the top surface are observed. As in the case of the steel plate, these currents arise due to curling of induced currents on the bottom surface around the plate's edges. Moreover, in Figure S6 these currents around the edge are clearly seen, too. However, total current on the top surface of the aluminum plate is lower in the figure than that observed in Figure S3 for the steel plate. One of the reasons behind this is coupling of the induced currents on the bottom surface to the top surface. Since these currents are in reverse direction with currents arisen by curling of bottom surface currents around the plate edges and currents emerged with looping magnetic fields produced by the coil, currents on the top surface diminish.

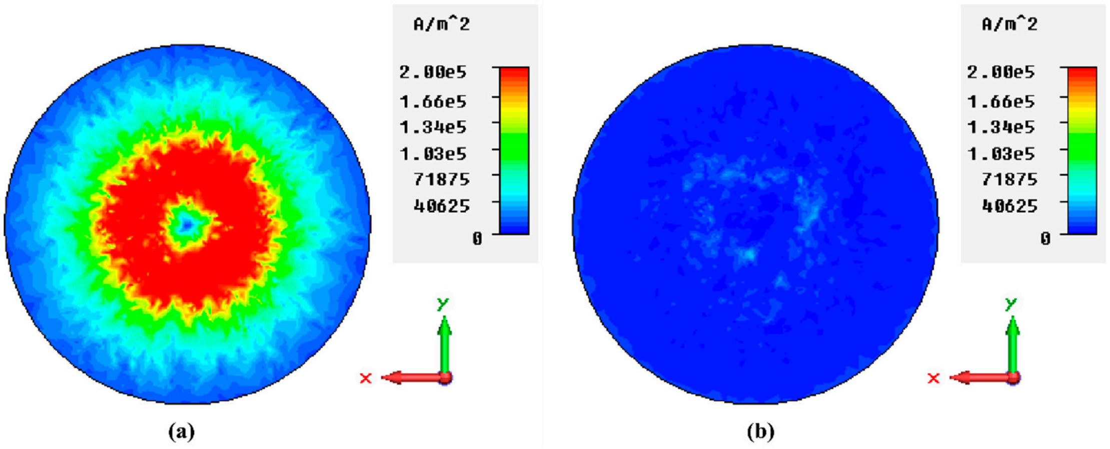

Figure S1. (**a**) Tangential current amplitude distribution on bottom surface of the steel plate; and (**b**) on top surface of the steel plate. Here plate and the coil centers coincide, *i.e.*, coil is covered by the plate's projection completely.

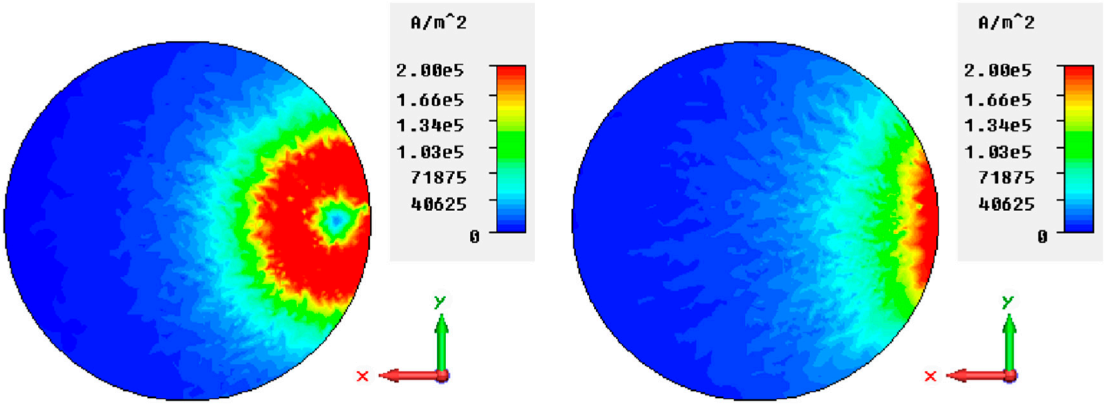

(a) (b)

Figure S2. (**a**) Tangential current amplitude distribution on bottom surface of the steel plate; and (**b**) on top surface of the steel plate. Here horizontal distance between the plate and the coil centers is 75 mm, *i.e.*, approximately 70% of the coil is covered by the plate's projection.

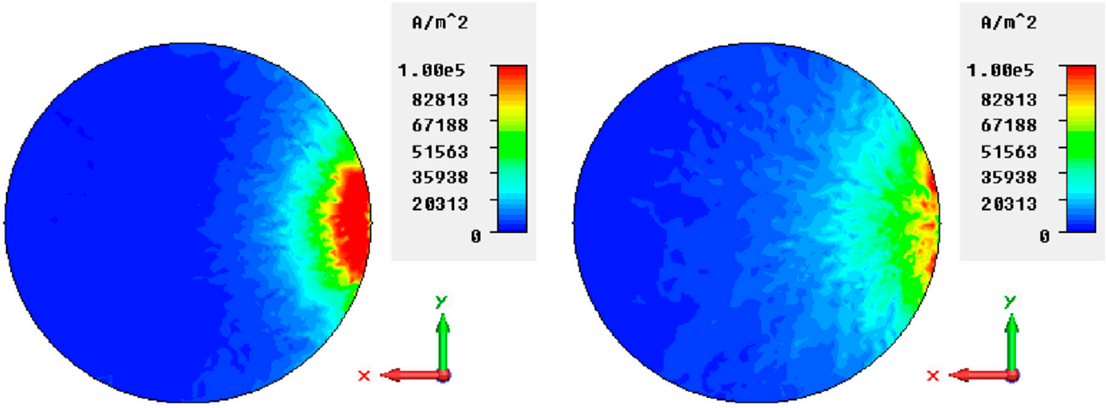

(a) (b)

Figure S3. (**a**) Tangential current amplitude distribution on bottom surface of the steel plate; and (**b**) on top surface of the steel plate. Here horizontal distance between the plate and the coil centers is 115 mm, *i.e.*, approximately 10% of the coil is covered by the plate's projection.

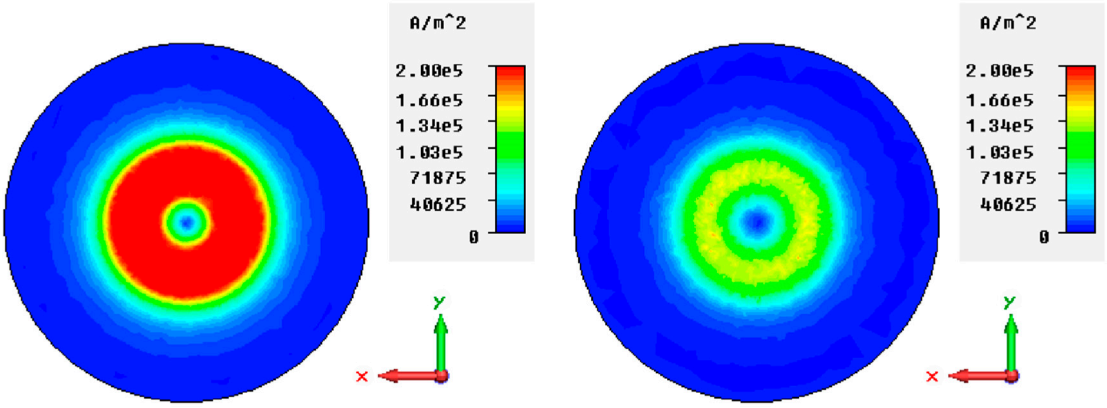

(a) (b)

Figure S4. (**a**) Tangential current amplitude distribution on bottom surface of the aluminum plate; and (**b**) on top surface of the aluminum plate. Here plate and the coil centers coincide, *i.e.*, coil is covered by the plate's projection completely.

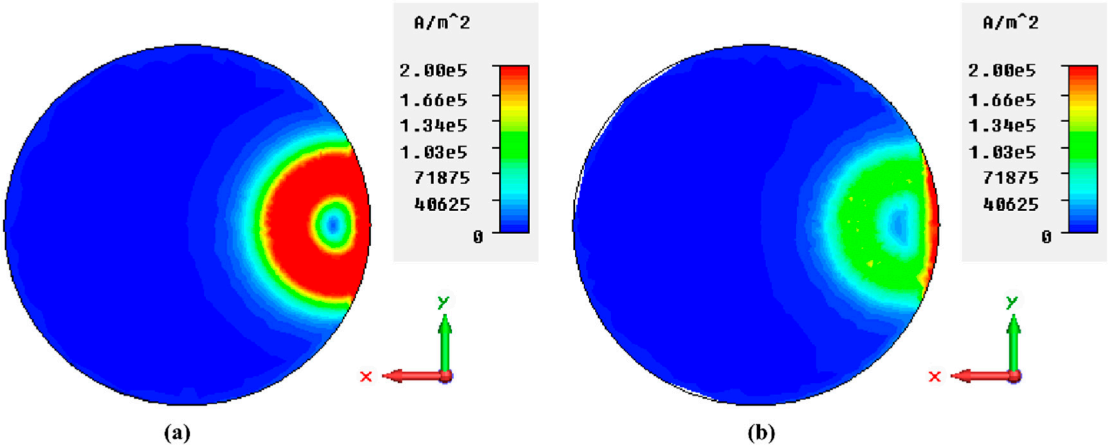

Figure S5. (**a**) Tangential current amplitude distribution on bottom surface of the aluminum plate; and (**b**) on top surface of the aluminum plate. Here horizontal distance between the plate and the coil centers is 75 mm, *i.e.*, approximately 70% of the coil is covered by the plate's projection.

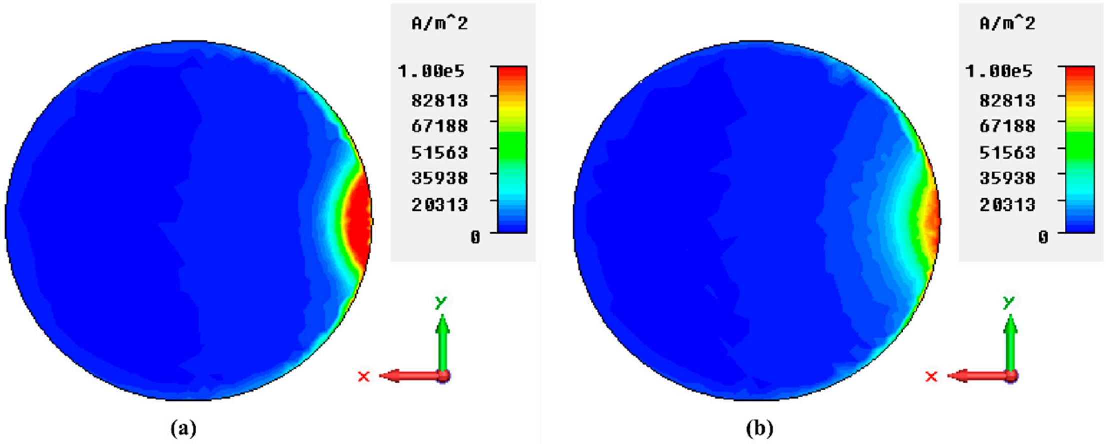

Figure S6. (**a**) Tangential current amplitude distribution on bottom surface of the aluminum plate; and (**b**) on top surface of the aluminum plate. Here horizontal distance between the plate and the coil centers is 115 mm, *i.e.* approximately 10% of the coil is covered by the plate's projection.
